# Evaluation of biochemical evidence suggestive of subclinical myocardial injury using high-sensitivity cardiac troponin T in patients with prediabetes: a cross-sectional study

**DOI:** 10.7717/peerj.21716

**Published:** 2026-09-24

**Authors:** Nur Syakirin Nor Ariffin, Tuan Salwani Tuan Ismail, Wan Nor Fazila Hafizan Wan Nik, Aniza Mohammed Jelani, Najib Majdi Yaacob, Nani Draman, Masliza Yusoff, Wan Mohd Saifuhisam Wan Zain

**Affiliations:** 1 Department of Chemical Pathology School of Medical Sciences, Universiti Sains Malaysia, Kota Bharu, Kelantan, Malaysia; 2 Hospital Pakar Universiti Sains Malaysia, Universiti Sains Malaysia, Kota Bharu, Kelantan, Malaysia; 3 Unit of Biostatistics and Research Methodology School of Medical Sciences, Universiti Sains Malaysia, Kota Bharu, Kelantan, Malaysia; 4 Department of Family Medicine School of Medical Sciences, Universiti Sains Malaysia, Kota Bharu, Kelantan, Malaysia; 5 Klinik Kesihatan Bandar, Ministry of Health Malaysia, Kota Bharu, Kelantan, Malaysia

**Keywords:** Prediabetes, Myocardial injury, Troponin, Cardiac biomarker, Chemical pathology, Clinical chemistry, Primary care, Diabetes, High-sensitivity troponin T, Laboratory medicine

## Abstract

**Background:**

Growing evidence indicates that cardiovascular abnormalities may develop during the prediabetic period. One of the earliest signs of these is biochemical evidence suggestive of subclinical myocardial injury characterised by elevated high-sensitivity cardiac troponin levels (>the 99th percentile upper reference limit). There is a paucity of studies on high-sensitivity troponin T (hs-cTnT) levels, the prevalence of subclinical myocardial injury, and related cardiometabolic predictors in individuals with prediabetes in Southeast Asia. Therefore, this study aimed to determine the distribution of hs-cTnT values suggestive of subclinical myocardial injury; assess the relationship between hs-cTnT values and HbA1c levels; and identify the predictors of suggestive of subclinical myocardial injury in people with prediabetes in Kota Bharu, Kelantan, Malaysia.

**Methods:**

This cross-sectional study included 101 adults with prediabetes at two primary care centres in Kota Bharu, Kelantan, Malaysia. Blood samples were collected for the evaluation of hs-cTnT, HbA1c, lipid profile (total cholesterol, triglycerides, high- and low-density lipoprotein cholesterol), urea, and creatinine levels. Biochemical evidence suggestive of subclinical myocardial injury was defined as hs-cTnT concentrations >14.00 ng/L. Associations between hs-cTnT values and HbA1c levels were analysed using Spearman’s rank correlation; predictors suggestive of subclinical myocardial injury were evaluated using Firth bias-corrected logistic regression.

**Results:**

The median hs-cTnT value was 7.29 ng/L (interquartile range: 3.85–12.33). Biochemical evidence suggestive of subclinical myocardial injury was detected in 14.9% (*n* = 15) of participants. Hs-cTnT values in participants suggestive of myocardial injury ranged from 14.71 to 149.20 ng/L. No significant association was found between hs-cTnT values and HbA1c levels (Spearman’s r = −0.053; *p* = 0.602). In exploratory Firth bias-corrected multivariable logistic regression analysis, higher systolic blood pressure was independently associated with biochemical evidence suggestive of subclinical myocardial injury after adjusting for demographic, metabolic, and cardiovascular risk factors (adjusted odds ratio: 1.13per mmHg increase, 95% confidence interval: 1.05–1.24; *p* = 0.001).

**Conclusions:**

Biochemical evidence suggestive of subclinical myocardial injury was present in 14.9% of adults with prediabetes, possibly associated with elevated systolic blood pressure. No significant association was found between hs-cTnT values and HbA1c levels. These findings suggest that myocardial injury may occur early during prediabetes. A larger population-based study would help verify these findings and determine the clinical significance of subclinical myocardial injury in this patient population.

## Introduction

Prediabetes is a transitional metabolic condition between normoglycaemia and type 2 diabetes mellitus. Malaysian clinical practice guidelines for the management of type 2 diabetes mellitus (Malaysian Ministry of Health, 2020) define prediabetes as glycated haemoglobin (HbA1c) levels of 5.7–<6.3%. WHO/ADA guidelines cite HbA1c levels of 5.7–6.4% (American Diabetes Association, 2019). A slightly lower cutoff was adopted for the Malaysian population due to lower cutoff values for the diagnosis of diabetes mellitus (a HbA1c level of 6.3%). These cutoffs demonstrated higher sensitivity in the Malaysian population (Wan Nazaimoon et al., 2013).

Growing evidence suggests that prediabetes leads to high cardiovascular morbidity and mortality prior to the development of diabetes mellitus (Cai et al., 2020; Siopis, Scibilia & Kiat, 2023). Cardiovascular disease is one of the leading causes of death worldwide. Hyperglycaemia below the diagnostic criteria for diabetes has been shown to cause adverse cardiovascular outcomes through endothelial dysfunction, oxidative stress, chronic low-grade inflammation, insulin resistance, and haemodynamic stress (Selvin et al., 2014). Evidence from large population-based studies indicates that cardiovascular injury begins early in the dysglycaemic spectrum, with people with prediabetes demonstrating subclinical myocardial damage and early vascular abnormalities (Selvin et al., 2014).

Dysglycaemia represents a significant public health burden in Malaysia; the reported prevalence of diabetes mellitus was 18.3% in adults in 2019 and nearly half of affected individuals remain undiagnosed. Prediabetes has been identified in 11.6% of the Malaysian population, and the percentage of people with prediabetes progressing to diabetes over a lifetime is estimated to be up to 70% (Akhtar et al., 2022; Institute for Public Health (IPH), National Institutes of Health, Ministry of Health Malaysia, 2020; Cai et al., 2020). Malaysia is one of the countries with the highest prevalence of diabetes in Asia, constituting a considerable cardiometabolic burden at the population level. Importantly, accumulating evidence suggests that cardiovascular risk associated with prediabetes occurs independently of progression to diabetes, underscoring the need for early cardiovascular risk assessment in individuals with prediabetes (Cai et al., 2020).

High-sensitivity cardiac troponin (hs-cTnT) assays have allowed the identification of low-grade myocardial injury which could not be detected in the past with conventional methods. Cardiac troponin is a structural protein of the cardiomyocyte contractile apparatus; concentrations above the 99th percentile upper reference limit are associated with increased morbidity. Elevated hs-cTnT values in asymptomatic patients have been demonstrated to be one of the predictors of heart failure and cardiovascular mortality (Selvin et al., 2014).

Overall, large population-based cohort studies, including the Atherosclerosis Risk in Communities (ARIC) study and the Multi-Ethnic Study of Atherosclerosis, have demonstrated that subclinical myocardial injury and unrecognized myocardial infarction are more prevalent in individuals with prediabetes with elevated troponin values (Selvin et al., 2014). However, these associations are generally modest and typically detectable only in very large population samples. Moreover, the relative contributions of glycaemic and non-glycaemic factors, such as blood pressure, to myocardial injury in prediabetes remain incompletely understood. In addition, reversion from a prediabetic to normoglycaemic state is associated with a reduction in cardiovascular and all-cause mortality risk in the Asian population. Thus, this should be considered in guidelines for the prevention of type 2 diabetes as well as cardiovascular morbidity and mortality in populations worldwide (Liu et al., 2021). Early identification of individuals at risk of cardiovascular morbidity and mortality is therefore even more relevant. Consistent with these observational findings, recent *post-hoc* analyses of the US Diabetes Prevention Program Outcomes Study (DPPOS) and the Chinese DaQing Diabetes Prevention Outcomes Study (DaQingDPOS) showed that participants who achieved prediabetes remission to normoglycaemia through structured lifestyle intervention had achieved approximately a 50% reduction in the risk of cardiovascular death or hospitalisation for heart failure (adjusted HR 0.47 in DPPOS and 0.49 in DaQingDPOS) with benefits persisting over two to three decades of follow-up. These landmark intervention studies demonstrate that remission from prediabetes is not only associated with improved prognosis but also represents a potentially modifiable treatment target for diabetes prevention and reduction of cardiovascular risk (Arreola et al., 2026).

Although the international literature on this topic has expanded, data on subclinical myocardial injury detected using hs-cTnT in individuals with prediabetes in Malaysia and other Asian populations remain limited. Population-specific studies are required given the high prevalence of cardiometabolic risk factors and the use of HbA1c as a key diagnostic marker of dysglycaemia in clinical practice. Therefore, this study aimed to (1) determine the distribution of hs-cTnT values suggestive of subclinical myocardial injury (2) determine the proportion of biochemical evidence suggestive of subclinical myocardial injury (3) assess the relationship between hs-cTnT values and HbA1c levels and (4) identify the predictors suggestive of subclinical myocardial injury in adults with prediabetes in Kota Bharu, Kelantan, Malaysia. To our knowledge, this is the first study assessing myocardial injury in adults with prediabetes in Malaysia.

## Materials and Methods

### Study design and setting

This cross-sectional study was conducted between June 2024 and May 2025 at two primary care centres in Kota Bharu, Kelantan, Malaysia: an outpatient clinic in Hospital Pakar Universiti Sains Malaysia (HPUSM), and a government health clinic in Kota Bharu. These centres treat various adult populations and provide routine screening and follow-up for metabolic disorders such as prediabetes.

### Study population

Adult patients with prediabetes aged between 18 and 59 years at HPUSM outpatient clinic or Kota Bharu government health clinic were recruited between 1 June 2024 and 31 May 2025. Malaysian clinical practice guidelines were used to define prediabetes (HbA1c levels of 5.7–<6.3%) (Malaysian Ministry of Health, 2020).

### Inclusion criteria

Adults aged 18–59 years with prediabetes, defined as HbA1c levels of 5.7–<6.3%, under follow-up at HPUSM outpatient clinic or Kota Bharu government health clinic during the study period were included.

### Exclusion criteria

Individuals with diabetes mellitus, those receiving glucose-lowering therapy, current smokers, pregnant or breastfeeding women, and those with pre-existing cardiovascular or renal disease were excluded to minimise confounding, particularly by factors known to influence cardiac troponin concentrations. Pre-existing diabetes mellitus, cardiovascular or renal diseases were excluded based on medical records. Renal disease also was excluded based on renal function indices (serum urea and creatinine) in which any participants with elevated urea and/or creatinine will be excluded from the study.

### Sample size estimation

Sample size was estimated according to the study objectives. For the estimation of hs-cTnT values, the required sample size was determined using an online mean estimator (https://najibmy.netlify.app/tools/sample-size/mean/estimate-mean/). Based on previously published data reporting a median hs-cTnT value of 13.00 ng/L (interquartile range (1QR) 8.2–21.6) in individuals with prediabetes, the standard deviation (SD) was estimated as 13.71 using an estimated mean–SD conversion in R. With a 95% confidence level and a margin of error of 5 units, the minimum sample size required was 29 participants; after adjusting for an anticipated 20% attrition rate, the corrected sample size was 35 participants.

For analysis of the relationship between hs-cTnT and HbA1c, sample size was determined using G*Power (version 3.1.9) for bivariate correlation analysis. Assuming a moderate effect size (r = 0.30), a two-sided alpha of 0.05, and 80% power, the required sample size was 84 participants. After accounting for an anticipated 20% attrition rate, the final required sample size was 101 participants. Accordingly, 101 individuals with prediabetes were recruited and the sample size was sufficient for all the objectives of this study.

The sample size calculation did not assume that the reported median hs-cTnT concentration was the mean. The source study reported the sample median, first quartile, third quartile, and sample size. Therefore, the Box–Cox method, implemented using the *bc.mean.sd()* function in the *estmeansd* package in R, was used to estimate the corresponding sample mean and SD. The Box–Cox method identifies a power transformation under which the transformed distribution is assumed to be approximately normal. The mean and SD are estimated on the transformed scale and inverse-transformed to the original hs-cTnT scale. Therefore, the estimated SD of 13.71 ng/L was used only as an anticipated variability parameter for *a priori* sample-size planning; it was not interpreted as evidence that hs-cTnT follows a normal distribution on its original scale.

### Data collection

Participants who met the inclusion criteria were selected randomly and invited to enrol in the study. Initially, 400 patients with prediabetes were screened for eligibility. After applying the selection criteria, 101 patients were invited by phone. Selected patients were screened based on medical records at HPUSM outpatient clinic and Kota Bharu government health clinic. The records included demographic information (age, sex, ethnicity) and medical history (including any pre-existing diabetes and cardiovascular or renal diseases).

Blood pressure measurements were collected using standardised procedures: participants were given a minimum of 5 min of rest prior to measurement as blood pressure is a well-known predictor of myocardial stress and cardiac troponin release (Selvin et al., 2014). Height, weight, body mass index (BMI), and waist circumference were measured using calibrated equipment. Weighing scales and a stadiometer were used to measure body weight and height; BMI was then calculated as kg/m^2^. Waist circumference was measured at the midpoint between the iliac crest and the lowest palpable rib in cm with standard measuring tape. A total of 6 mL venous blood was collected from each participant using standardised procedures. Of this, 3 mL was transferred into an ethylenediaminetetraacetic acid (EDTA) tube for HbA1c measurement and 3 mL into a plain tube for the measurement of hs-cTnT, lipids, urea, and creatinine.

### Laboratory analysis

Hs-cTnT values were measured using a COBAS e 801 immunoassay analyser (Roche Diagnostics, Basel, Switzerland). The 99th percentile upper reference limit was 14.00 ng/L with a coefficient variation of less than 10%. HbA1c levels were determined using a Capillary 3 Octa analyser (Sebia). The capillary electrophoresis method of measurement by the analyser was standardised against the reference measurement procedure for HbA1c by the International Federation of Clinical Chemistry and Laboratory Medicine. A COBAS e 503 chemistry analyser (Roche Diagnostics) was used to evaluate lipid profiles (total cholesterol, triglycerides, high- and low-density lipoprotein cholesterol), serum urea, and creatinine. All analysers demonstrated acceptable analytical performance and underwent method verification procedures and regular internal quality control checks in accordance with ISO 15189 standards.

### Statistical analysis

Statistical analyses were performed using SPSS version 29. Continuous variables were assessed for normality; non-normally distributed variables are presented as median and IQR. Spearman’s rank correlation was used to assess the relationship between hs-cTnT and HbA1c due to non-normal distribution of troponin values. Univariable logistic regression analyses were performed to evaluate the association between participant characteristics and elevated hs-cTnT concentrations (>14 ng/L). Variables selected *a priori* based on established cardiovascular risk factors and biological plausibility were included in a multivariable model. Firth bias corrected logistic regression was applied to address potential small sample bias and risk of separation due to the limited number of outcome events (Selvin et al., 2014). A *p*-value < 0.05 was considered statistically significant.

### Ethics approval

Ethical approval for this study was granted by the Human Research Ethics Committee of Universiti Sains Malaysia (JEPeM USM code: USM/JEPeM/KK/24010070) and the Medical Research & Ethics Committee Ministry of Health Malaysia (NMRR ID: 24-00509-FHS). All participants provided written informed consent prior to enrolment.

## Results

A total of 101 adults with prediabetes were included in the analysis. The mean (SD) participant age was 47.3 (7.0) years, with a slight female predominance (53.5%); most participants were of Malay ethnicity (80.2%). Baseline demographic characteristics, blood pressure measurements, anthropometric indices, and laboratory parameters are summarised in Table 1.

**Table 1:** Baseline characteristics of prediabetic participants (*n* = 101).

Variable	Value
^a^Age (years)	47.3 ± 7.0
^b^Sex	
Female	54 (53.5)
Male	47 (46.5)
^b^Ethnicity	
Malay	81 (80.2)
Chinese	19 (18.8)
Indian	1 (1.0)
^a^SBP (mmHg)	112.7 ± 7.6
^a^DBP (mmHg)	76.4 ± 4.2
^a^BMI (kg/m^2^)	24.8 ± 3.0
^a^Waist circumference (cm)	91.7 ± 11.2
^a^Triglycerides (mmol/L)	1.3 ± 0.8
^a^Total cholesterol (mmol/L)	5.1 ± 1.1
^a^HDL cholesterol (mmol/L)	1.1 ± 0.3
^a^LDL cholesterol (mmol/L)	3.4 ± 1.0
^a^Urea (mmol/L)	4.9 ± 1.6
^a^Creatinine (µmol/L)	78.6 ± 23.7

**Note:**

Data were expressed as mean ± SD for a and *n* (%) for b.

The study population was generally normotensive, with a mean (SD) systolic blood pressure (SBP) of 112.7 (7.6) mmHg and diastolic blood pressure (DBP) of 76.4 (4.2) mmHg. Mean (SD) BMI and waist circumference were 24.8 (3.0) kg/m^2^ and 91.7 (11.2) cm, respectively. According to Asian-specific classification criteria, a BMI ≥ 23 kg/m^2^ is considered overweight (Malaysian Ministry of Health, 2023; WHO Expert Consultation, 2004), placing the average BMI of this cohort within the overweight range. Recommended waist circumference cutoffs for central obesity in Asian populations are ≥90 cm for men and ≥80 cm for women (International Diabetes Federation, 2006). The mean waist circumference in this prediabetic cohort suggests an increased burden of central adiposity and elevated cardiometabolic risk. Lipid profile parameters were modestly elevated; renal function indices (urea and creatinine) were within normal ranges.

**Figure 1:**
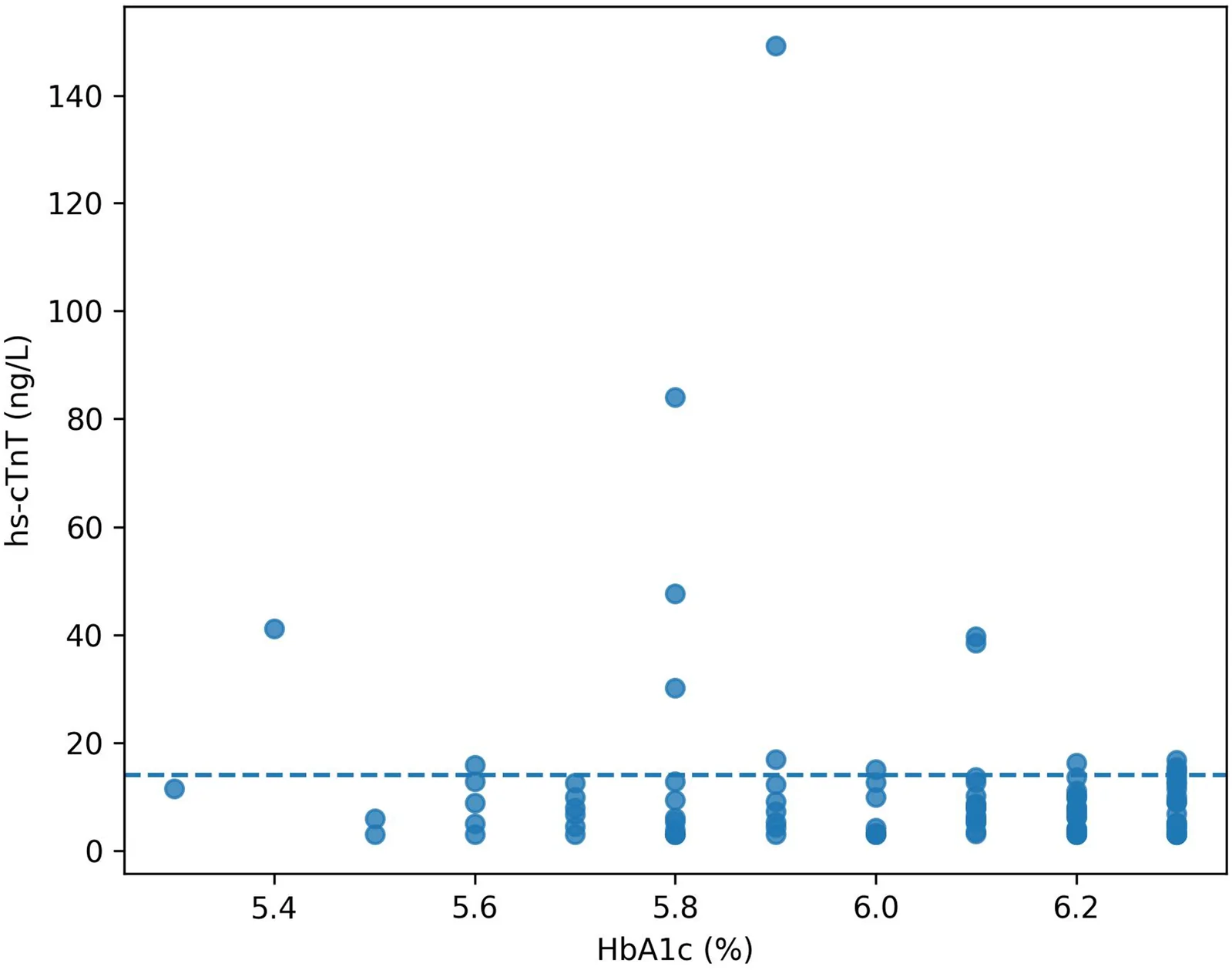
Relationship between hs-cTnT values and HbA1c levels in prediabetes.

The median hs-cTnT value (whole cohort) was 7.29 ng/L (IQR 3.85–12.33), with values ranging from 3.00 to 149.20 ng/L (Table 2). Biochemical evidence suggestive of subclinical myocardial injury, defined as hs-cTnT values >14.00 ng/L, was identified in 14.9% (*n* = 15) of participants (Table 3); these values ranged from 14.71 to 149.20 ng/L (Table 2). No significant association was found between hs-cTnT values and HbA1c levels (Spearman r_s_ = −0.053, *p* = 0.602) (Fig. 1).

**Table 2:** Distribution of h s-cTnT values among prediabetic participants.

Statistic	hs-cTnT (ng/L)
Median (IQR)	7.29 (3.85–12.33)
Range	
All participants	3.00–149.20
Participants with myocardial injury (hs-cTnT > 14 ng/L)	14.71–149.20

**Table 3:** Proportion of prediabetic participants with normal and elevated hs-cTnT.

hs-cTnT category	*n* (%)
≤14 ng/L	86 (85.1)
>14 ng/L	15 (14.9)

**Note:**

Normal hs-cTnT was defined as hs-cTnT ≤14 ng/L and presence of myocardial injury was defined as hs-cTnT >14 ng/L.

In the univariable logistic regression analysis, SBP, DBP, and BMI were associated with elevated hs-cTnT (>14.00 ng/L). A multivariable Firth bias-corrected logistic regression model incorporating age, sex, race, HbA1c, triglycerides, high- and low-density lipoprotein cholesterol, BMI, SBP, and DBP, only SBP remained independently associated with hs-cTnT values >14.00 ng/L following adjustment. Each 1 mmHg increase in SBP was associated with a 13.0% increase in the odds of subclinical myocardial injury (adjusted odds ratio: 1.13; 95% confidence interval: 1.05–1.24; *p* = 0.001) (Table 4).

**Table 4:** Factors associated with subclinical myocardial injury using Firth bias-corrected logistic regression analysis.

Variable	Crude OR (95% CI)	*p*-value	Adj. OR (95% CI)	*p*-value
Age	0.99 [0.91–1.08]	0.842		
Sex				
Female	Reference			
Male	1.04 [0.33–3.26]	0.943		
Race				
Malay	Reference			
Non-Malay	0.42 [0.04–1.90]	0.285		
HbA1c	0.52 [0.06–5.16]	0.557		
TG	0.86 [0.31–1.73]	0.710		
HDL	2.09 [0.28–14.76]	0.464		
LDL	0.86 [0.47–1.50]	0.596		
BMI	1.38 [1.11–1.76]	0.003		
SBP	1.13 [1.05–1.24]	0.001	1.13 [1.05–1.24]	0.001
DBP	1.24 [1.08–1.46]	0.002		

**Note:**

SBP remained independently associated with subclinical myocardial injury after adjustment using Firth bias-corrected multivariable logistic regression (adjusted odds ratio 1.13; 95% confidence interval 1.05–1.24; *p* = 0.001)

## Discussion

There was substantial heterogeneity in myocardial injury burden in this prediabetic cohort (median hs-cTnT: 7.29 ng/L [IQR 3.85–12.33]; range: 3.00–149.20). Biochemical evidence suggestive of subclinical myocardial injury (hs-cTnT values > 14.00 ng/L) was identified in 14.9% of participants (range: 14.71–149.20 ng/L). This is comparable to findings from large population-based cohorts: in the ARIC study, approximately 10–15% of individuals with HbA1c-defined prediabetes had hs-cTnT values above the 99th percentile, associated with increased risk of heart failure and mortality (Selvin et al., 2014).

Similarly, Witkowski et al. (2021) reported that individuals with prediabetes and detectable hs-cTnT values in a large Cleveland Clinic cohort had significantly higher risk of adverse cardiovascular outcomes than those with undetectable troponin values. Although the study evaluated detectable hs-cTnT rather than values exceeding the 99th percentile upper reference limit, higher values were independently associated with adverse outcomes. Collectively, these findings support the growing evidence that myocardial injury may be present at early stages of the dysglycaemic spectrum, prior to diabetes mellitus or clinically apparent cardiovascular disease (Selvin et al., 2014; Cai et al., 2020). However, as this study did not include a normoglycaemic comparator group, it cannot determine whether elevated hs-cTnT concentrations are more prevalent than might be expected in individuals without prediabetes.

Notably, the median hs-cTnT value in this study (7.29 ng/L) was lower than that reported by Witkowski et al. (2021), who found a median value of approximately 13.00 ng/L in individuals with prediabetes. This discrepancy may reflect variations in study design and population characteristics, including recruitment from a primary care setting, ethnic differences, smaller sample size, and the exclusion of participants with known cardiovascular/renal disease. These factors likely reduced the overall burden of myocardial injury while permitting detection of clinically meaningful subclinical changes. The wide range of hs-cTnT values observed in this cohort may suggest that myocardial injury in prediabetes is not uniform and may reflect contributions from haemodynamic stress, metabolic abnormalities, and individual susceptibility (de Lemos et al., 2010; Ndumele et al., 2016; Brannick & Dagogo-Jack, 2018).

SBP emerged as the sole independent predictor for biochemical evidence suggestive of subclinical myocardial injury following adjustment for demographic, metabolic, and cardiovascular risk factors. Elevated SBP increases myocardial wall stress, impairs coronary microvascular perfusion, and promotes cardiomyocyte stretch, resulting in chronic low-grade myocardial injury detectable by high-sensitivity troponin assays (Selvin et al., 2014; Ndumele et al., 2016). Notably, the study population was generally normotensive (mean SBP 112.7 ± 7.6 mmHg). Therefore, the observed association may reflect subtle variations in SBP within the normal range rather than overt hypertension. This suggests that even modest increases in SBP may contribute to myocardial stress in individuals with prediabetes.

Prior studies have shown that hs-cTnT is more strongly associated with haemodynamic stressors, such as systolic hypertension and arterial stiffness, than traditional atherosclerotic risk factors, supporting a predominantly non-atherosclerotic mechanism of myocardial injury in early dysglycaemia (Ndumele et al., 2016; Brannick & Dagogo-Jack, 2018). However, given the limited number of participants with elevated hs-cTnT, the multivariable analysis should be interpreted cautiously. Although Firth-bias corrected logistic regression was used to minimise small-sample bias, the possibility of model instability and overfitting cannot be excluded. Thus, the findings should be considered exploratory. Sensitivity analyses of the influence of very high hs-cTnT values were not performed; thus, it is possible that the observed association between SBP and elevated hs-cTnT may have been influenced by markedly elevated hs-cTnT values.

The participant with the highest hs-cTnT value had a markedly abnormal lipid profile characterised by elevated atherogenic lipid fractions. Dyslipidaemia contributes to coronary atherosclerosis and microvascular dysfunction, which may lead to chronic myocardial ischaemia and low-grade cardiomyocyte injury detectable by high-sensitivity troponin assays in the absence of acute coronary syndromes (Ndumele et al., 2016; Brannick & Dagogo-Jack, 2018). Population-based studies have similarly demonstrated that elevated hs-cTnT is more common in individuals with adverse cardiometabolic profiles, reflecting subclinical myocardial injury related to underlying coronary pathology (Selvin et al., 2014; Witkowski et al., 2021).

This study did not find a significant association between hs-cTnT values and HbA1c levels. This could be because associations may be too subtle to achieve statistical significance in smaller cross-sectional studies. Thus, the absence of statistical significance is likely attributable to limited statistical power given the modest sample size and relatively small number of myocardial injury events. Studies with larger cohorts have shown that incremental increases in glycaemic exposure are associated with progressive elevations in myocardial injury markers (Tabák et al., 2012; Gao et al., 2021).

Participants with cardiovascular disease, renal disease, pregnancy, diabetes mellitus, current smokers, and those receiving glucose-lowering therapy were excluded. Individuals with cardiovascular disease may exhibit chronically elevated hs-cTnT values due to ongoing myocardial ischaemia or structural heart disease (de Lemos et al., 2010; Thygesen et al., 2018). Renal disease was excluded as impaired glomerular filtration is associated with reduced troponin clearance and chronic hs-cTnT elevation (Giannitsis et al., 2010). Smoking promotes oxidative stress, endothelial dysfunction, and prothrombotic states which may contribute to troponin release (Brannick & Dagogo-Jack, 2018). Pregnancy is associated with physiological haemodynamic changes which may influence circulating hs-cTnT. Exclusion of individuals with diabetes ensured that observed troponin elevations reflected myocardial injury within the prediabetic stage, not advanced diabetic vascular complications (Selvin et al., 2014; Cai et al., 2020). These exclusions were to help ensure that any hs-cTnT elevations observed represent subclinical myocardial injury attributable to early dysglycaemia.

Careful consideration was given to pre-analytical, analytical, and post-analytical processes. Blood sampling was standardised to minimise haemolysis and collection artefacts. All hs-cTnT measurements used a high-sensitivity assay with a coefficient of variation <10% at the 99th percentile upper reference limit. All analysers demonstrated acceptable internal and external quality control performance and underwent verification in accordance with ISO 15189 standards. Electronic result transmission *via* the laboratory information system eliminated manual transcription errors. No laboratory errors were identified, including in samples with elevated hs-cTnT values.

All participants with hs-cTnT concentrations above the 99th percentile upper reference limit were referred for cardiology evaluation in accordance with routine clinical practice. Further assessments including clinical evaluations, electrocardiography, transthoracic echocardiography, and functional testing are essential to exclude occult coronary artery disease or silent ischaemia (Neumann et al., 2019; Thygesen et al., 2018). Persistently elevated hs-cTnT concentrations in asymptomatic individuals with normal initial investigations–defined as the absence of ischaemic changes and regional wall motion abnormalities on echocardiography, and no clinical features of acute coronary syndrome–have been previously described and are thought to reflect chronic myocardial injury related to haemodynamic stress or subclinical coronary disease rather than acute myocardial infarction (Giannitsis et al., 2010; Thygesen et al., 2018).

From a clinical perspective, the presence of subclinical myocardial injury in a substantial proportion of individuals with prediabetes highlights the importance of early cardiovascular risk stratification in this population. Cardiovascular risk in prediabetes appears to be mediated not only by glycaemic burden but also by haemodynamic and metabolic stressors, underscoring the importance of comprehensive risk factor optimisation, particularly blood pressure and lipid management (Cai et al., 2020; Siopis, Scibilia & Kiat, 2023).

This study has several limitations. The cross-sectional design precludes causal inference, the modest sample size limits statistical power, and the absence of a normoglycaemic comparator group limits interpretation of the prevalence estimates. Although elevated hs-cTnT concentrations were observed in 14.9% of participants, this study cannot determine whether this prevalence is higher than might be expected in a similar cohort without prediabetes. Nevertheless, this study provides valuable data from a Malaysian population and contributes to the growing evidence that elevated hs-cTnT concentrations may be present during the prediabetic stage (Selvin et al., 2014; Cai et al., 2020).

### Future directions

A large, multicentre, population-based prospective study with longitudinal follow-up would help determine whether elevated hs-cTnT concentrations in individuals with prediabetes predict incident cardiovascular events. Serial measurements of both hs-cTnT and high-sensitivity cardiac troponin I (hs-cTnI) may provide additional insights, as these assays differ in analytical characteristics, biological variation, and prognostic performance. Comparative evaluation of hs-cTnT and hs-cTnI may help clarify whether myocardial injury patterns observed in prediabetes are assay-specific or reflect broader pathophysiological mechanisms.

## Conclusions

Biochemical evidence suggestive of subclinical myocardial injury was identified in 14.9% of individuals with prediabetes in the absence of evident cardiovascular disease, potentially associated with elevated SBP. No significant association was observed between hs-cTnT concentrations and HbA1c. These findings support the concept that myocardial injury may occur during the prediabetic stage. Early cardiovascular risk assessment with particular emphasis on blood pressure optimisation may therefore be important in individuals with prediabetes.

## Supplemental Information

10.7717/peerj.21716/supp-1 Supplemental Information 1 Raw data.

10.7717/peerj.21716/supp-2 Supplemental Information 2 Codebook for raw data (gender and race).

10.7717/peerj.21716/supp-3 Supplemental Information 3 STROBE Checklist.

## Additional Information and Declarations

### Data Availability

The following information was supplied regarding data availability:

The data is available in the Supplemental Files.
