# Supplementary material for: Evaluation of biochemical evidence suggestive of subclinical myocardial injury using high-sensitivity cardiac troponin T in patients with prediabetes: a cross-sectional study
Source: PeerJ. 2026 Sep 24;14:e21716. doi: 10.7717/peerj.21716 (PMC13616196; doi:10.7717/peerj.21716)
Supplement: Supplementary file 1 [file peerj-14-21716-s003.pdf]

STROBE Statement—Checklist of items that should be included in reports of ***cross-sectional studies***

	Item No	Recommendation	Remark (where item addressed)
**Title and abstract**	1	(*a*) Indicate the study's design with a commonly used term in the title or the abstract	Study design (cross-sectional) was addressed in the title.
		(*b*) Provide in the abstract an informative and balanced summary of what was done and what was found	Study method and study results were addressed in the abstract.
**Introduction**			
Background/rationale	2	Explain the scientific background and rationale for the investigation being reported	Introduction Paragraph 1 to 5
Objectives	3	State specific objectives, including any prespecified hypotheses	Introduction Paragraph 5
**Methods**			
Study design	4	Present key elements of study design early in the paper	Materials and method section.
Setting	5	Describe the setting, locations, and relevant dates, including periods of recruitment, exposure, follow-up, and data collection	1.Study design and setting section. 2.Study population section. 3.Data collection section.
Participants	6	(*a*) Give the eligibility criteria, and the sources and methods of selection of participants	1. Inclusion and exlcusion criteria section. 2. Data collection section.
Variables	7	Clearly define all outcomes, exposures, predictors, potential confounders, and effect modifiers. Give diagnostic criteria, if applicable	1. Outcomes (median value of hs-cTnT, proportion of myocardial injury etc) were addressed in results section. Potential confounders addressed in exlcusion criteria and explained in discussion section paragraph 7. Predictors was identified using logistic regression addressed in

			statistical analysis section.<br>2. Diagnostic criteria for prediabetes was mentioned in inclusion criteria section. Diagnostic criteria for myocardial injuty was mentioned in results section paragraph 4
Data sources/ measurement	8*	For each variable of interest, give sources of data and details of methods of assessment (measurement). Describe comparability of assessment methods if there is more than one group	Sources of data were addressed in data collection and laboratory analysis section.
Bias	9	Describe any efforts to address potential sources of bias	Potential sources of bias (small-sample bias) was addressed in statistical analysis section.
Study size	10	Explain how the study size was arrived at	Sample size estimation section.
Quantitative variables	11	Explain how quantitative variables were handled in the analyses. If applicable, describe which groupings were chosen and why	Data collection and laboratory analysis section.
Statistical methods	12	(*a*) Describe all statistical methods, including those used to control for confounding	Statistical analysis section.
		(*b*) Describe any methods used to examine subgroups and interactions	Not applicable
		(*c*) Explain how missing data were addressed	Sample size estimation section-20% attrition rate.
		(*d*) If applicable, describe analytical methods taking account of sampling strategy	Not applicable
		(*e*) Describe any sensitivity analyses	Not applicable
**Results**			
Participants	13*	(a) Report numbers of individuals at each stage of study—eg numbers potentially eligible, examined for eligibility, confirmed eligible, included in the study, completing follow-up, and analysed	Results section paragraph 1 (total number of participants)
		(b) Give reasons for non-participation at each stage	Not applicable. 100% subject recruitment based

			on sample size estimation.
		(c) Consider use of a flow diagram	Not applicable
Descriptive data	14*	(a) Give characteristics of study participants (eg demographic, clinical, social) and information on exposures and potential confounders	Demographic addressed in results section paragraph 1. Participant with confounders were excluded-refer exclusion criteria section.
		(b) Indicate number of participants with missing data for each variable of interest	No missing data.
Outcome data	15*	Report numbers of outcome events or summary measures	Results section
Main results	16	(*a*) Give unadjusted estimates and, if applicable, confounder-adjusted estimates and their precision (eg, 95% confidence interval). Make clear which confounders were adjusted for and why they were included	Confounders were excluded stated in exclusion criteria section and also addressed in discussion section paragraph 7.
		(*b*) Report category boundaries when continuous variables were categorized	Boundaries were reported in Mean (SD) and Median (IQR) in results section and table.
		(*c*) If relevant, consider translating estimates of relative risk into absolute risk for a meaningful time period	Not applicable
Other analyses	17	Report other analyses done—eg analyses of subgroups and interactions, and sensitivity analyses	Correlation between hs-cTnT and HbA1c and logistic regression analysis were reported in results section.
**Discussion**			
Key results	18	Summarise key results with reference to study objectives	Discussion section paragraph 1,2,4,6
Limitations	19	Discuss limitations of the study, taking into account sources of potential bias or imprecision. Discuss both direction and magnitude of any potential bias	Discussion section paragraph 11
Interpretation	20	Give a cautious overall interpretation of results considering objectives, limitations, multiplicity of analyses, results from similar studies, and other relevant evidence	Discussion section paragraph 2, 3, 4,5, 6,

Generalisability	21	Discuss the generalisability (external validity) of the study results	Discussion section paragraph 8- all analysers underwent routine internal and external quality control.
**Other information**			
Funding	22	Give the source of funding and the role of the funders for the present study and, if applicable, for the original study on which the present article is based	Acknowledgements section.

*Give information separately for exposed and unexposed groups.

Note: An Explanation and Elaboration article discusses each checklist item and gives methodological background and published examples of transparent reporting. The STROBE checklist is best used in conjunction with this article (freely available on the Web sites of PLoS Medicine at http://www.plosmedicine.org/, Annals of Internal Medicine at http://www.annals.org/, and Epidemiology at http://www.epidem.com/). Information on the STROBE Initiative is available at www.strobe-statement.org.
